# The Human Cell Atlas: Technical approaches and challenges

**DOI:** 10.1093/bfgp/elx029

**Published:** 2017-10-28

**Authors:** Chung-Chau Hon, Jay W Shin, Piero Carninci, Michael J T Stubbington

**Affiliations:** 1 RIKEN Center for Life Science Technologies, Division of Genomic Technologies, Yokohama, Kanagawa, Japan; 2 Wellcome Trust Sanger Institute, Wellcome Genome Campus, Hinxton, Cambridge, UK

**Keywords:** Human Cell Atlas, single cell, RNA sequencing, bioinformatics

## Abstract

The Human Cell Atlas is a large, international consortium that aims to identify and describe every cell type in the human body. The comprehensive cellular maps that arise from this ambitious effort have the potential to transform many aspects of fundamental biology and clinical practice. Here, we discuss the technical approaches that could be used today to generate such a resource and also the technical challenges that will be encountered.

## Introduction

**Figure 1.**
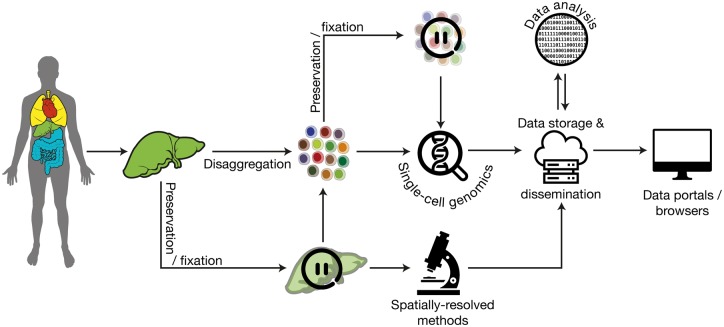
Overview of the paths from tissue acquisition to data dissemination in the HCA. scRNAseq protocols act on disaggregated suspensions of cells from human organs with optional stages at which samples may be fixed or otherwise preserved. Spatially resolved methods analyse sections of fixed tissues. The data that are generated must be stored, analysed and disseminated.

The Human Cell Atlas (HCA) is a large, international consortium that aims to identify and describe every cell type in the human body [1]. The comprehensive cellular maps that arise from this ambitious effort have the potential to transform many aspects of fundamental biology and clinical practice. It is now possible to consider creating such a resource because of the explosive proliferation of techniques that explore biology at the resolution of individual cells and thus are able to capture the true variation present within complex cell populations. An effort of this magnitude will present many technical challenges throughout the journey from tissue acquisition to data dissemination (Figure 1). Although all the steps in this process are achievable with current technologies, there is still huge scope for the optimization of existing methods and the development of innovative new approaches at every stage.

The exact approach that will be taken to build the HCA remains under discussion by all of those involved in the initiative and such decisions will be communicated through channels outside of this review. Here, we discuss the current state-of-the-art of technical approaches that could be used to generate the Atlas in three areas: sample acquisition, data-generating technologies and computational analyses. The HCA is likely to ultimately measure many different aspects of the cells that it studies, but we feel that two foundational approaches will be (1) single-cell RNA sequencing (scRNAseq) and (2) understanding the physical arrangement of cells within organs and tissues through the analysis of spatially resolved gene expression at single-cell resolution. scRNAseq can be used to define the molecular identities of a large number of cells at affordable costs and is a sufficiently mature and distributed technology to be available to a diverse range of laboratories worldwide. Although spatially resolved methods are less mature and well-distributed, identifying the spatial relationships of cells in complex tissues will produce a true atlas that links basic genomics with clinical pathology. Here, we focus on these two approaches to allow us to survey existing technologies and to examine the challenges that remain.

## Sample acquisition

An atlas of human cells starts with an obvious challenge: obtaining samples from all the tissues that are present in a human. This is, of course, significantly more difficult than the acquisition of equivalent samples from model organisms and, furthermore, the tissues must be suitable for use in experiments that characterize all the cell types that are present. Previous large-scale projects that aimed to characterize gene expression across diverse cell types include the Genotype-Tissue Expression Project (GTEx; [2]) and FANTOM5 [3]. However, a major difference between studies on bulk populations of cells and the single-cell resolution that will be a defining feature of the HCA is that previous projects were able to fix, freeze or lyse tissues immediately after collection and then ship the samples to central facilities for gene expression assays. Current standard scRNAseq protocols typically require the use of freshly isolated cells, and, moreover, it is imperative that the transcriptomes of the cells are not allowed to decay between acquisition and processing. This will ensure that the observed cell-type-specific transcriptional identities are biologically relevant.

Post-acquisition RNA degradation has been shown to affect RNA sequencing (RNA-seq) data leading to non-random and transcript-dependent changes in apparent gene expression [4, 5]. The influence of post-mortem ischaemia on RNA-seq was also observed in the GTEx project, where ischaemic time accounted for 40% of variance in RNA quality [2]. Thus, the HCA will need to use tissue acquisition strategies that minimize the ischaemic interval between collection and processing of each sample. Three modes of tissue collection are particularly suited to minimizing ischaemic time. First, biopsies from living donors allow tissue to be collected and processed rapidly but are restricted in the range of organs that can be sampled. Collection of tissue from donors who are undergoing surgery can obtain samples from organs that are resected or from non-involved tissues (often skin) but, again, this is limited to a subset of all organs within the body. Finally, a close partnership with organ donation networks and transplant surgeons provides a strategy that minimizes ischaemic time but permits collection of samples from, potentially, all organs. Here, consent is obtained to procure samples for research from deceased subjects who are donating organs for transplant. In the typical case of donation after brainstem death, confirmation of death is followed by anaesthesia and preparation of organs, whilst the donor remains ventilated. Ventilation is then withdrawn and the donor is immediately perfused with cold organ preservation solution, which reduces cell metabolism whilst also mitigating against the potential damage caused by the cold temperatures [6–8]. This method of acquisition has been used successfully in other studies that required fresh human samples [9–11] and, we believe, holds great promise for permitting the HCA to map all human tissues.

A requirement for cells to be processed immediately after collection reduces the complexity of experiments that can be designed and limits the geographical distance between sites of sample collection and cell processing. Overcoming this limitation would be of great value in enabling the HCA to maximize efficiencies and to extend the range of potential donors. There is understandable interest in the development of methods that can preserve cells for storage before later downstream processing. Cell preservation can occur by two means: cryopreservation or chemical fixation.

Kere and colleagues [12] used freezing to preserve endometrial biopsies before scRNAseq analysis and, although they reported good results for stromal cells, data from epithelial cells were poor. Experiments using high-throughput droplet microfluidics found that data from cryopreserved peripheral blood mononuclear cells (PBMCs) were comparable with those from fresh cells [13]. In addition, biological insights could be gained from frozen bone marrow aspirates when healthy donors were compared with donors undergoing treatment for acute myeloid leukaemia, although these samples were not compared with equivalent fresh cells. Work from the Heyn laboratory has shown that cryopreservation maintains transcriptomic profiles of cell line suspensions, PBMCs and tumour samples [14]. This is promising, although there is evidence that, in some cases, the cryopreservation procedure biases the recovery of certain cell populations.

The cryopreservation methods described here used either biopsies or dissociated cell suspensions. In the latter case, this would require dissociation of tissues before preservation. For the case of sample acquisition during organ donation, it would be ideal if entire tissue pieces could be preserved without the need for additional manipulations, as this would minimize the burden on collection networks. Recent work found that hypothermic preservation of whole mouse kidneys in organ preservation solution (as discussed in the context of donor perfusion above) maintains transcriptome stability for up to 3 days [15]. This approach is appealing, although further work is required to show that this is generalizable to a variety of human tissues and to understand the maximum storage times that are possible for each tissue type. Chemical fixation of dissociated cell suspensions before scRNAseq has been demonstrated for cells from model organisms using fixation with formaldehyde [16] or methanol [17,18] and for human embryonic stem cells and glia using formaldehyde [19]. An advantage of fixation methods is that they permit the use of split–pool indexing to uniquely label the complementary DNA (cDNA) generated from each cell rather than requiring the capture of separate individual cells [16,18]. This can dramatically reduce the cost per cell and so permit higher throughput.

Whilst some groups work to optimize the collection, preservation and processing of tissues and cells for use in scRNAseq protocols, others have developed methods that require only intact single nuclei. These protocols permit the use of frozen tissues or those, such as brain, where stringent dissociation can adversely affect data quality in individual cells. Quantification of mRNA transcripts solely from within nuclei appears to provide sufficient information to elucidate the transcriptional states of individual cells and has been performed on single nuclei that were partitioned (in order of increasing cell throughput) by micromanipulation [20], microfluidic capture [21], fluorescence-activated cell sorting (FACS) [22, 23] and droplet capture [24].

The preservation and sequencing methods discussed here have great potential to support the success of the HCA by increasing the flexibility of experiments that can be performed. However, the diverse methods and species that have been used to validate the various approaches serve to emphasize that we lack a systematic understanding of the performance characteristics of each protocol in human tissue. This would be very informative in designing optimal processes, pipelines and experiments for the HCA.

Two additional points are absolutely critical no matter what methods are used to acquire and process the tissue samples. First, the collection of detailed, extensive and accurate metadata will be essential to ensure that each experiment can be analysed and interpreted correctly. These metadata must include details about the donor’s medical status, the procedures and methods used to collect the samples and any relevant time intervals (such as that between cessation of ventilation and sample collection). In addition, detailed information must be recorded about the protocols used for all sample preservation and processing. Secondly, it would be unthinkable to collect samples for the HCA without adhering to the necessary legal and ethical requirements that control work with human tissues. Procedures must be put in place to ensure that work within the HCA meets all of the relevant requirements in the country in which it is performed. This will be complex [25] but key to the success of the project.

## Data-generating technologies

Once tissue samples have been acquired, they must be analysed to determine the cell populations contained within. The choice of platforms and protocols used within the HCA will depend on balancing requirements of throughput, data quality and cost. scRNAseq platforms are becoming ever more prevalent and diverse. A key driver of the rapid growth in single-cell research has been the commercial availability of instruments that partition and process cells for scRNAseq analysis. The first of its kind was Fluidigm’s C1 platform, which captures cells at low to medium throughput (96 or 800) using a microfluidic circuit, where the cells are lysed and reverse transcribed, and cDNA is amplified. When using its 96-cell chip, this method provides sequencing coverage over the entire length of each transcript, which can provide information beyond simple gene expression estimation [26]. Furthermore, custom protocols can be implemented on the microfluidics device, and several research groups have adapted their own ‘ex-chip’ protocols [27, 28] making it possible to share and run identical protocols in multiple laboratories.

Similar data to those generated by the C1 platform can be acquired by deposition of individual cells into microtitre plates either by FACS [29] or nano-dispensers such as Wafergen’s ICELL8 [30], where sequencing libraries can then be generated by hand or with the use of liquid-handling robotics. A highly robotized pipeline can process thousands of cells in a day using these methods, although high reagent volumes (when compared with microfluidic methods) mean that this is a more expensive approach.

The HCA will require unbiased, broad surveys of the cells that are present in human tissues. Therefore, scRNAseq methods that permit large numbers of cells to be analysed affordably in a single experiment will be crucial. Droplet-based platforms generate an emulsion of nanolitre-volume aqueous compartments within a flow of oil. Each droplet forms a reaction chamber that can encapsulate a single cell with the potential to capture thousands of cells in a run. The Drop-seq and inDrop [31, 32] instruments can be assembled using readily available equipment, and this approach is attractive to many laboratories. However, standardization of the assembled apparatus and quality control of reagents is essential, particularly when intending to integrate data into a larger effort such as the HCA. Commercially available droplet instruments such as the Chromium (10X Genomics) or ddSeq (Illumina/Biorad) platforms are also available and remove the need for self-assembly albeit with higher cost per cell. However, commercial platforms are typically limited to the manufacturer’s scRNAseq kit precluding customizations or novel protocols. Nonetheless, innovation in single-cell platforms continues. Just in the past year, Shalek and colleagues [33] introduced Seq-Well, where single cells are captured in an array of ∼86 000 subnanolitre wells along with the same uniquely indexed beads as in DropSeq. Seq-Well provides a simple and portable platform for massively parallel scRNAseq with the potential to disseminate the arrays to multiple data collection sites, including clinical and rural surroundings.

Advances in DNA sequencing technologies also provide novel ways to sequence the transcriptome from individual cells. Long-read sequencing using the PacBio instrument allows the profiling of RNA isoforms expressed from individual genes [34]. Single-cell profiling of VLMC-2 cells identified about 2000 unique transcripts mapped to around 700 genes and 1000 distinct isoforms. The Oxford Nanopore MinION sequencing technology (ONT) is a portable device based on single-molecule sequencing technology that provides long reads by performing voltage-driven molecule translocations through small nanosensors [35]. Using mouse B1a cells, the ONT RNA-seq has been used to analyse full-length cDNA samples derived from single cells and identified and quantified novel isoforms at the single-cell level [36]. However, these methods currently provide significantly lower read output (and thus lower single-cell throughput) than methods using short-read technology: the studies described here analysed only six and seven single cells, respectively. This currently limits their utility for the HCA.

Gene expression is not the only way to define cell states and so single-cell measurements at the genomic and epigenomic levels will be useful in the HCA. Existing methods can profile DNA sequence [37], chromatin accessibility [38], chromatin state [39], three-dimensional (3D) architecture [40, 41] and methylation status [42]. ‘Multi-omics’ approaches combine one of these methods with scRNAseq to provide even deeper information about cell state by simultaneously assessing, for example genome sequence and RNA expression (G&T-seq; [43]), DNA methylation and RNA expression (scMT-seq; [44]) or cell surface proteins and RNA expression (CITE-seq; [45]).

The HCA will not only generate a catalogue of cell types using scRNAseq but will also create a true atlas by elucidating the spatial relationships between cells in the context of tissues. This will require methods that quantify the expression of genes or proteins in a spatially resolved way. One such method is single-molecule RNA fluorescent *in situ* hybridization (smFISH) [46, 47], which makes gene expression measurements that are highly accurate and well correlated with those from DropSeq and Fluidigm scRNAseq platforms. Gene dropout rates, measured by Gini coefficient, were higher in sequencing platforms than in RNA-FISH [48]. Several adaptations of RNA-FISH have been introduced to increase the number of target RNAs that can be detected in a single experiment: SeqFISH [49] and MER-FISH [50]. These hybridization-based methods require probes to a previously selected panel of genes and so do not provide coverage of the entire transcriptome. Other spatially resolved methods do not require a priori target selection and, instead, use artificial nucleotide sequences to encode spatial coordinates within an RNA-seq library generated from a tissue section [51] or direct RNA-seq from tissue sections and whole-mount embryos [52]. Finally, computational frameworks have been developed to infer spatial coordinates by comparison with existing *in situ* gene expression data [53, 54]. High-resolution methods for the detection by mass spectrometry of proteins bound by heavy metal-labelled antibodies have also been described [55, 56].

Existing work using scRNAseq has shown that these techniques can reveal important and novel biological insights; current techniques will permit the initial construction of the HCA. However, there remains room for improvement, optimization and technical development. Current scRNAseq platforms exhibit high levels of technical noise [57], and the efficiency of capture of RNA molecules remains relatively low. Quantitative assessment suggested a capture efficiency of 5–60% [58], and these inefficiencies are attributed to biases in molecular capture (e.g. template switching; reverse transcription) and amplification. Increases in efficiency will enable us to profile the cellular composition of tissues at ever increasing levels of detail. Continued work is required to optimize the efficiency of reverse transcription and polymerase chain reaction and to understand how to best use unique molecular identifiers (UMIs), or spike-in reference mRNAs to discriminate technical noise from biological variation. Furthermore, existing droplet-based scRNAseq methods sequence short tags from the 3′ end of mRNA molecules and so do not capture information from the entire length of the message. A strategy to capture and profile the complete transcriptome (and not just polyadenylated RNAs) would permit quantification of lowly abundant and important regulatory RNAs such as enhancer RNAs, long non-coding RNAs and miRNAs that account for large fractions of the human transcriptome [59]. In fact, a recently developed method based on RNA ligation and oligonucleotides specifically masking ribosomal RNAs successfully profiled miRNAs in single cells [60]. Efforts to increase the resolution and throughput of spatially resolved methods will further enhance their value to the HCA as will additional dissemination of such methods to laboratories worldwide.

We do not believe that any single method that will be suitable for the entirety of the HCA. Different approaches are complementary and should be applied in combination to provide data that can be integrated to generate a complete atlas. A deep and systematic understanding of the performance and cost characteristics of each method would help to develop a set of best practice guidelines and minimal quality standards to inform experimental design. The ultimate technology for the HCA would be a platform that can deeply profile unbiased and spatially resolved gene expression in thousands of single cells with high precision at low cost. However, absent such a method, the initial efforts construct the atlas will drive technology development and inform the community as to the best ways to profile tissue composition at this scale. It will be crucial to be sufficiently flexible so as to assess and implement suitable new methods, as they become available to ensure that the atlas is generated using the best available technologies.

## Computational analyses

The major challenges of analysing scRNAseq are its high dimensionality (i.e. many genes in many cells) and high variability (i.e. noise). Genuine biological variation is combined with technical noise including dropouts and amplification biases. Furthermore, the HCA is likely to analyse millions of cells that are processed in batches across different locations and at different times, and thus batch effects must be carefully considered. The computational challenges can be split into four broad areas: (1) estimation of expression levels, (2) definition of cell identity, (3) identification of gene signatures and (4) analysis of spatially resolved data. Finally, in the context of the HCA, large data sets could be unified and integrated into ensemble analyses.

### Estimation of expression levels

Before estimation of gene expression from scRNAseq data, quality control must be performed. Some ‘cells’ within the data in fact represent captured debris, free-floating RNA or are otherwise of low quality, and these should be excluded from downstream analyses. Quality control metrics such as gene detection, mapping rates or apparent expression of mitochondrially encoded genes can be used to identify low-quality cells [61–63] and, although some tools [64] provide convenient ways to visualize various quality control metrics, the choices of thresholds remain arbitrary. More recently, statistical methods integrating multiple metrics have been developed to identify low-quality cells in a data-driven manner [65–67].

Following quality control, raw gene expression is normalized, so that relative expression levels are comparable between cells. Normalization strategies used in bulk RNA-seq typically involve a global scaling factor for all genes and all samples, which is not suitable for scRNAseq [68]. To address this, a number of tools use simple statistical models along with the detection of spike-ins at known concentrations to inform normalization [69, 70], while other recently developed tools use more complex Bayesian approaches based on cell-specific noise estimated from spike-ins [71–73]. Others approaches model cell-specific factors without spike-ins, and these approaches can be valuable in droplet-based scRNAseq, where it is not possible to include spike-ins along with each cell. These methods can attempt to learn the properties of clusters of similar cells, instead of considering each cell independently [74, 75] or explore gene-specific scaling, on the basis that a global scaling factor might lead skewed estimations for weakly or highly expressed genes [76, 77]. Alternatively, to accommodate dropouts, tools have been developed to impute missing values under gene-specific dropout models [78, 79].

Even after normalization, other confounders, notably batch effects [80] and biological factors such as the cell cycle [81], may still obscure the signal of interest. Methods originally developed to correct batch effects in microarrays have been applied to meta-analyses of scRNAseq data [82] and, more recently, batch correction methods specifically designed for scRNAseq have also been reported [83, 84]. In addition to batch effects, heterogeneity because of both technical noise and biological variation can complicate analyses. In cycling cells, assessment and removal of the variation caused by the cell cycle can help to reveal other important biological processes [81, 85] and, more generally, sources of variation can be decomposed into technical and a variety of biological factors [86].

The HCA is likely to generate scRNAseq data at an unprecedented scale and thus integrate data sets generated from many different samples by a diverse set of laboratories. Thus, a unified and optimized set of methods for quality control, normalization and removal of confounding factors would allow analyses to be performed across the entire set of HCA data. A list of tools used for addressing these questions is summarized in Table 1.

**Table 1.** Tools for estimation of expression levels

Goals	Methods/features	Tools
Quality control	Visualizing various quality control metrics	Scater [64]
	Data-driven identification of low-quality cells	SinQC [65], Cellity [66], SCell [67]
UMI processing	General processing of UMI	umis [87]
	Systematically correct UMI sequencing errors	UMI-tools [88]
Normalization with spike-in	Simple statistical models	SAMstrt [69], GRM [70]
	Bayesian approaches to normalize cell-specific noises	BASiCS [71], BEARscc [72], TASC [73]
Normalization without spike-in	Estimating cell-specific factors by learning the properties of clusters of similar cells	scran [74], BISCUIT [75]
	Gene-specific scaling	SCnorm [76], Census [77]
	Imputation with gene-specific dropout models	SCONE [78], MAGIC [79]
Batch effect removal	Originally developed for microarrays or bulk RNA-seq but used in scRNAseq	Combat [89], RUV [78]
	Specifically developed for scRNAseq	scPLS [83], BatchEffectRemoval [84]
Cell cycle effect removal	Remove the cell cycle components from the expression values	scLVM [81]
	Identify and remove the genes that are affected by cell cycle stages	ccRemover [85],
Simulation	Simulation of scRNAseq data sets for benchmarking methods	Splatter [90], powsim [91]

### Definition of cell identity

To describe and define every cell type in the human body, one must first address the meaning of ‘cell type’. It will not be trivial to arrive at such a definition that is generally applicable to the data sets generated for the HCA. One working conceptual framework is that a cell’s identity at a given moment is defined by the unique combination of all the factors that influence it [92]. In this framework, a cell type (e.g. hepatocyte) can be considered as the stable and permanent features of its identity, whilst a cell state can be considered as the transient aspects of its status (e.g. an immune cell response to cytokines). We expect that an important use of the large HCA data set is likely to be in developing these concepts through the construction of data-driven and generalizable mathematical definitions of cell type and state.

In practice, it is likely that there will be multiple ways in which one could define terms such as these depending on the exact types of data that are used (e.g. scRNAseq only, multi-omics or spatially resolved data). Importantly, multiple definitions do not have to be mutually exclusive and could all provide utility in addressing different biological questions.

Here, we will address the concrete case of defining cell types and states using scRNAseq data sets. This is typically achieved by first performing a dimensionality reduction step to project a high-dimensional matrix of gene expression values into a lower-dimensional space [93]. This is followed by a clustering step to assign cells to distinct groups such that cells within a group are sufficiently transcriptionally similar to each other to be usefully referred to as a cell type. Principal component analysis (PCA) has been extensively used in scRNAseq studies, although its assumption of linearity [93] is often not met by these data sets. Non-linear methods such as t-distributed stochastic neighbour embedding (t-SNE [94]), non-negative matrix factorization [95, 96] and diffusion maps [97, 98] have also been applied. Other dimensionality reduction algorithms specifically model or impute dropouts [99–101]. Recently, a machine learning approach, which learns a custom distance metric that best fits the data, was shown to outperform many other model-based dimension reduction methods [102].

In most workflows, a clustering step is performed on the reduced-dimension data to assign cells to distinct clusters. Traditionally, this has been *k*-means or hierarchical clustering, although, recently, the application of graph theory-based methods has also proved useful [103, 104]. Some workflows perform standard dimension reduction (e.g. PCA and t-SNE) and clustering (e.g. *k*-means) algorithms in combinations (agglomeratively or iteratively) to improve robustness [102–107]. A number of techniques classify cell types without dimensionality reduction, mitigating against the risk of losing biologically relevant signal [108, 109] and, in some cases, also allow cells to have partial memberships in multiple clusters [110]. Other methods are specifically intended to discriminate rare cell types [111, 112].

As the HCA will cover a wide range of tissues containing cell populations of various complexities, it is unlikely that one clustering method would fit all scenarios and so the performance of clustering methods should be objectively benchmarked. Assigning cells to discrete clusters is not appropriate when describing cell populations with continuous phenotypes (i.e. cell states), e.g. stem cells during differentiation and immune cells during activation [113]. In these cases, cells can be represented as points along a continuum [114], and cells participating in such trajectories will be observed within the HCA and will require methods to analyse them. Owing to the stochasticity of each cell’s temporal progression in a dynamic process, a snapshot of a pool of cells captures cells at various stages along their trajectory. Thus, the temporal ordering of each cell, i.e. pseudotime, can be estimated [115]. Currently, >20 tools have been developed for trajectory inference (Table 2) and their methodologies have been recently reviewed [115]. These tools can be broadly classified into two categories based on whether they assume a linear trajectory or permit branching. It should be noted that trajectory inference can be applied to both time-stamped data sets (e.g. *in vitro* differentiation time series) and snapshot data sets (e.g. a mixture immune cells from blood). Within the HCA, trajectory inference methods should be chosen to best fit the biological context.

**Table 2.** Tools for definition of cell identity

Goals	Methods/features	Tools
Dimensionality reduction	Linear, PCA	PCA [93]
	Non-linear, t-SNE embedding	t-SNE [94]
	Nonlinear, diffusion map	destiny [97]
	Nonlinear, non-negative matrix factorization	Nimfa [95], NMFEM [96]
	Linear, specifically designed to model, or to impute, dropouts	ZIFA [99], ZINB-WaVE [100], CIDR [101]
	Machine learning for a custom distance metric	SIMLR [102]
Classification of cell types	Graph theory-based clustering methods	SNN-cliq [103], PhenoGraph [104]
	Combinations of standard dimension reduction and clustering algorithms	pcaReduce [105], ICGS [107], SC3 [106], Seurat [53]
	Bi-clustering of cells and genes	BackSPIN [109]
	Hierarchical clustering on centred Pearson’s correlation	SINCERA [108]
	Grade of membership models	CountClust [110]
	Distinguish rare cell types from background noises	RaceID [111], GiniClust [112]
Trajectory inference	Linear trajectory inference	DeLorean [116], embeddr [117], pseudogp [118], SCENT [119], SCIMITAR [120], SCORPIUS [121],Waterfall [122], WaveCrest [123]
	Branched trajectory inference	BEAM [77], CellTree [124], DPT [125], ECLAIR [126], FORKS [127], GPfates [113], k-branches [128], MFA [129], Monocle [130], Mpath [131], Ouija [132], PHATE [133], SCOUP [134], scTDA [135], SCUBA [136], SLICE [137], SLICER [138], Slingshot [139], StemID [140], TASIC [141], Topslam [142], TSCAN [143], Wanderlust [144], Wishbone [145]

### Identification of gene signatures

Defining the gene signatures specific to particular cell types or states allows us to build classifiers for cell identity prediction and to draw conclusions about the differentiation mechanisms and functions of the cells of interest. In addition, a reduced set of gene signatures is crucial to inform the design of probe-based methods that measure gene expression in a spatial context [109]. The most common approach to detect gene signatures is to identify genes that are differentially expressed between cell types or states. However, the strong overdispersion and dropouts of scRNAseq data are not adequately accommodated by most methods developed for bulk RNA-seq, as these methods generally assume a unimodal distribution of gene expression, which violates the bimodal distribution of expression levels in scRNAseq. To address this, a number of single cell-specific methods have been developed [146–149]. Whilst these methods test for significant differences between mean expression levels, other methods were developed to detect the differences in the distribution of expression levels [150, 151]. In some scenarios, genes that vary during continuous transitions across cell states, rather than between distinct cell types, are of interest. These can be detected by methods that identify genes expression changes along inferred cell trajectories [130, 152].

Genes are often expressed in a coordinated way (i.e. co-expressed) as part of the processes that underlie biological functions and so gene signatures of cell types and states can also be investigated using gene regulatory networks (GRNs) [153]. The scale of the HCA will provide an opportunity to learn GRNs across multiple biological processes. Although many GRN inference algorithms are available [154] and most of them were not designed for scRNAseq, applications of these algorithms to single-cell data sets have been preliminarily explored [19, 154–161]. Binarized Boolean models represent the states of genes as 'on or off' and are relatively robust to the presence of dropouts. A Boolean network can then be created to describe the regulatory circuit of genes, based on the covarying patterns of their binary expression states [162–164]. However, a general drawback of Boolean models is that the dimension of its state space increases exponentially with the number of genes. Alternatively, some other methods exploit the temporal information of dynamic processes, i.e. pseudotime, to infer GRNs [165]. This is achieved in an *ad hoc* approach by computing the maximum correlation of all possible lags in the pseudotime scale and using maximum correlation to replace the traditional Pearson’s correlation for constructing a GRN [166]. It is also possible to take full advantage of temporal information by modelling the level of gene expression over the continuous pseudotime scale to identify co-expressed genes for GRN construction [120, 167]. A list of tools used for identification of genes signatures is summarized in Table 3.

**Table 3.** Tools for identification of gene signatures

Goals	Methods/features	Tools
Identification of differentially expressed genes	Detect the differences in mean of expression levels, by modelling the bimodal distribution of expression levels	MAST [146], BPSC [147], M3Drop [148], SCDE [149]
	Detect the differences in distribution, instead of mean, of expression levels	SCPattern [123], scDD [151], D3E [150]
	Identify variations in expression attributable to sets of genes	f-scLVM [86], PAGODA [149]
	Incorporate pseudotime information to identify gene significantly changed along the inferred cell trajectory	switched [152], monocle [130]
Identification of cell-type-specific genes	Signature genes co-identified during clustering of cells	BackSPIN [109], nimfa [95]
	Regression-based approaches	SINCERA [108]
	Machine learning approaches	SVM-RFE [168]
Inference of GRN	Originally developed for microarrays or bulk RNA-seq but used in scRNAseq	WGCNA [169], GENIE3 [170]
	Boolean network models specifically designed for single-cell data sets	SingCellNet [162], SCNC [163], BTR [164]
	Incorporate pseudotime information to identify co-expressed genes	LEAP [166], SCODE [167], SCIMITAR [120]

### Analysis of spatially resolved data

As discussed above, the HCA is likely to include spatially resolved data about gene or protein expression from cells within the context of their native tissues. These data sets will require appropriate analytical tools and methods of integration with scRNAseq data generated from dissociated cells.

The field of spatial methods is not as mature as that of scRNAseq, but there are reports showing the exciting potential of these approaches. Work in the mouse midbrain first used scRNAseq to identify distinct cell types and to define cell-type-specific genes [171]. The marker genes were then used to inform the choice of probes for smFISH such that each cell type could be identified within microscopy images of brain sections. Another study in the mouse liver performed scRNAseq in parallel with smFISH using probes for landmark genes already known to have diverse zonation patterns [172]. The sequencing and imaging data sets were combined by measuring smFISH signals for the landmark genes in nine spatial layers. Probabilistic inference was then used to assign each single cell to a layer according to the expression of the landmark genes within the scRNAseq data. In addition to methods that measure RNA levels, mass spectrometry-based detection of proteins has been used to investigate the spatial arrangement of cell types within tumours [55, 56].

The large scale of the HCA means that it will require automated methods for the analysis of spatially resolved data to address challenges such as the automated detection of cells and segmentation of images [55, 56, 173]. Once spatial gene expression patterns have been measured, it will be informative to identify genes whose expression varies within two-dimensional (2D) or 3D space (analogous to differential expression analysis in transcriptomic data). A recently reported method (SpatialDE) achieves this using a framework based on Gaussian process regression to classify genes with distinct spatial patterns [174].

### Ensemble analyses and data dissemination

One challenge presented by the scale and scope of the HCA will be how one should present the data derived from such a large number of cells. One possible approach would be to analyse the individual scRNAseq data sets generated from different tissues, i.e. groups of anatomically related cells, independently and then to integrate them into ensemble analyses. To manage thousands of millions of individual cells, novel methods and systems will need to be developed to group similar cells into manageable number (e.g. thousands) of conceptual meta-items, referred to as ‘meta-cells’. A meta-cell can be regarded as the consensus expression profile of its members (i.e. child-cells) from a distinct cell type or state. Meta-cells should be unique entities in the atlas and can be organized hierarchically, similar to a cell-type ontology [175] but defined in a data-driven manner. Meta-cells might be further organized by anatomical concepts [176], based on the physiological origins of their child-cells or spatial relationships in the context of tissues [53]. The consensus expression profiles of these meta-cells might be used as a reference panel to guide the analyses of scRNAseq data by, e.g. reference component analysis [177]. A global GRN might be constructed from all meta-cells for inferring gene signatures to groups of meta-cells, and the relationships between these meta-cells could be further visualized in a 2D or 3D space using existing visualization tools [97, 178–181].

## Conclusion

The HCA will use techniques and methods from exciting, fast-moving fields. This presents the project with a huge opportunity to drive technology development and to provide high-quality recommendations about best practice in a wide variety of areas. It is evident from the diversity of methods discussed above that systematic comparisons of method performance would enable the HCA community to ensure that approaches are chosen rationally in a data-driven manner. Initial work in this area has compared scRNAseq protocols using either published data sets on the basis of spike-in standards [87] or newly generated data sets on the same cell populations [57]. Benchmarking of computational methods for expression estimation, cell-type identification and trajectory inference is likely to require simulated data sets [90, 91].

Furthermore, we feel that it will be crucial to maintain flexibility and to consider new protocols, as they are developed to ensure that the HCA can take advantage of improvements in performance, cost or efficiency. Despite the challenges that lie ahead, this effort will not only be possible but will lead to a dramatic and valuable improvement in our understanding of human biology.

**Key Points**

- The HCA aims to identify and describe every cell type in the human body.
- Two main approaches to achieve this will be scRNAseq and spatially resolved methods.
- Sources of human tissue samples and appropriate handling techniques will be key to this project.
- Many single-cell sequencing approaches exist and so the HCA has the opportunity to perform systematic comparisons as well as to develop novel methods.
- Single-cell sequencing data present unique computations challenges and rich areas for innovation.

## Funding

This work was supported by a Research Grant from MEXT to the RIKEN Center for Life Science Technologies (to C.-C. H., J.W.S. and P.C.) and by Wellcome Trust Grant 206194 (to M.S.).

## Notes

**Chung-Chau Hon** is a research scientist at the RIKEN Center for Life Science Technologies focusing on the bioinformatic analyses of single-cell and FANTOM projects.

## Notes

**Jay W. Shin** is a team leader at the RIKEN Center for Life Science Technologies organizing single-cell research activities and FANTOM projects.

## Notes

**Piero Carninci** is a deputy director at the RIKEN Center for Life Science Technologies and the director of Division of Genomic Technologies. His research background is in technology development and noncoding RNA.

## Notes

**Michael J.T. Stubbington** leads the team at the Wellcome Trust Sanger Institute that is working on the Human Cell Atlas. His research interests include single-cell genomics and immune receptor repertoires.
